# Intracellular Tau Clearance by Immunotherapy Mediated by TREM21

**DOI:** 10.1002/alz.087717

**Published:** 2025-01-03

**Authors:** Rakez Kayed, Sagar Gaikwad

**Affiliations:** ^1^ University of Texas Medical Branch, Galveston, TX USA

## Abstract

**Background:**

Pathological tau aggregates cause cognitive decline in neurodegenerative tauopathies, including Alzheimer’s disease (AD), and more abundant in intracellular *vs*. extracellular compartments. However, current immunotherapies are slow and ineffective at clearing intracellular tau aggregates.

**Method:**

We developed toxic tau conformation–specific monoclonal‐antibody‐2–loaded micelles (TTCM2‐ms) that selectively recognize disease‐relevant tau aggregates in brain tissues from patients with AD, progressive supranuclear palsy, and dementia with Lewy bodies and potently inhibit tau‐seeding activity.

**Result:**

A single intranasal dose of TTCM2‐ms effectively cleared pathological tau, increased levels of synaptic proteins, and improved cognitive functions in aged tauopathy mice. Mechanistic studies suggest that TTCM2‐ms clears intracellular, synaptic, and seed‐competent tau aggregates via tripartite motif‐containing 21 (TRIM21), an intracellular antibody receptor and E3‐ubiquitin ligase. TRIM21 is essential for TTCM2‐ms–mediated clearance of tau pathology.

**Conclusion:**

Our findings suggest that intranasally administered TTCM2‐ms rapidly distributes across the brains of tauopathy mice. Further, TTCM2‐ms recognized and cleared pathological tau from the intracellular and synaptic compartments of neuronal cells via TRIM21, thus improving cognitive functions. This study provides insights into the mechanisms of an effective tau immunotherapy strategy against intracellular tau pathology in neurodegenerative tauopathies, including AD.

